# What do older adults think about involvement in health research through a citizen science lens? A situated qualitative study

**DOI:** 10.1186/s40900-026-00960-y

**Published:** 2026-10-30

**Authors:** Elaine Santana, Joana Bernardo, Rosa Silva, Fernanda Lanza, Iria Dobarrio-Sanz, Sílvia Silva

**Affiliations:** 1 University of Coimbra, Health Sciences Research Unit: Nursing (UICISA: E), Nursing School of the University of Coimbra (ESEUC), Rua Dr. José Alberto Reis, Coimbra, 3000-232 Portugal; 2 Center for Innovative Care and Health Tecnology (ciTechcare), School of Health Sciences, Polytechnic of Leiria, Leiria, Portugal; 3 Nursing School, University of Porto, RISE-Health, Nursing School, University of Porto, Porto, Portugal; 4 University of Coimbra, Health Sciences Research Unit: Nursing (UICISA: E); Federal University of São João Del-Rei., Minas Gerais, Brazil; 5 University of Coimbra, Health Sciences Research Unit: Nursing (UICISA: E);, Coimbra, Portugal; 6 University of Coimbra, Health Sciences Research Unit: Nursing (UICISA: E), Nursing School of the University of Coimbra (ESEUC), Coimbra, Portugal

**Keywords:** Citizen science, Older adults, Health research, Scientific research, Science communication

## Abstract

**Introduction:**

Citizen science (CS) has emerged as a promising approach to foster public involvement in the production of scientific knowledge, including in the field of health. However, for involvement to be effective, it is essential to examine the circumstances surrounding participation particularly from citizens’ perspectives in order to understand motivations, perceived benefits, and the conditions that may enable or hinder meaningful involvement in the process.

**Objective:**

To explore community-dwelling older adults’ perceptions and expectations regarding prospective involvement in health research through a citizen science lens.

**Methods:**

An exploratory, descriptive qualitative study was conducted using a focus group with community-dwelling older adults. Data were analysed using content analysis, supported by ATLAS.ti software.

**Results:**

Three themes were identified: (1) Between volunteering and science; (2) Becoming a future citizen scientist: identity, motivations, and perceived benefits; and (3) Becoming a future citizen scientist: challenges and conditions for involvement. Participants associated involvement with volunteering and social contribution, highlighting motivations such as contributing to society, staying active, and strengthening social ties. Anticipated benefits included lifelong learning, personal validation, and wellbeing. Key barriers included the need for training and ongoing support, technical language, and balancing involvement with personal routines. Role clarity, accessible communication, and alignment of expectations emerged as central conditions for effective involvement.

**Conclusion:**

This exploratory study provides preliminary and situated insights into how older adults perceive prospective involvement in health research through a citizen science lens. The findings suggest that future citizen science initiatives in health should make citizens’ roles, expectations and opportunities for influence explicit, while ensuring accessible communication, training, support, flexibility and recognition. These elements may help researchers move beyond symbolic involvement and support older adults in assuming a more confident and meaningful role in the research process.

**Supplementary Information:**

The online version contains supplementary material available at https://doi.org/10.1186/s40900-026-00960-y.

## Abstract (plain language summary)

**Why did we do this study?:**

Involving older adults in health research can help ensure research better reflects their needs. However, for this involvement to be active and meaningful, it is important to understand what people think about the idea, what motivates them, and what might make participation difficult.

**What did we do?:**

We spoke with eight community-dwelling older adults in a group discussion (focus group). The conversation was recorded, transcribed, and analysed to identify key ideas about involvement in research.

**What did we find?:**

Participants often understood “getting involved in research” as something similar to volunteering and supporting the community. They said they would be willing to take part in order to contribute to society, stay active and mentally involved, and strengthen social ties. They also mentioned benefits such as learning new things, feeling valued and useful, and improving wellbeing. To be actively involved, they felt it was important to have training and support, and for communication to be clear and simple, without overly technical language. They also emphasised the need for clearly defined roles and expectations, and flexibility to fit activities around routines and other commitments. Participants valued being recognised as “partners” or “collaborators”.

**What does this mean?:**

Our findings suggest that projects aiming to involve older adults should plan training and support, use accessible language, clarify citizens’ roles, and recognise their contributions, helping to make science more inclusive and closer to people.

**Supplementary Information:**

The online version contains supplementary material available at https://doi.org/10.1186/s40900-026-00960-y.

## Introduction

Citizen science (CS) has gained prominence as an innovative approach to scientific research by involving non-academic members of the public in different activities across the scientific process [1–5]. Within this approach, citizens may assume different levels of involvement [6, 7], participating, for example, in identifying relevant research questions, supporting recruitment, collecting data, developing and/or applying data collection instruments, as well as interpreting and disseminating findings. These contributions may occur in researcher-led projects, in which citizens act as advisors, collaborators or co-creators, or even in initiatives led by citizens themselves [1, 4, 6]. Rather than referring to a single fixed model, citizen science is described in the literature as a plural and evolving field, encompassing diverse participatory practices. In this manuscript, CS is used as a conceptual lens to emphasise citizens’ active contribution to research processes and knowledge production.

Although citizen science is well established in the natural and physical sciences [8, 9], in the health field this approach remains relatively recent and underexplored [3, 10, 11], despite its recognised promise and potential benefits. Its translation into health research also raises specific conceptual, methodological and ethical questions. In health-related projects, it is particularly important to distinguish between studies that merely collect data from citizens and initiatives in which citizens actively contribute to the production, interpretation or dissemination of scientific knowledge [1–4].

In recent years, there has been a growing movement towards shared clinical decision-making, with greater patient involvement in shaping treatment plans. Although this shift does not automatically translate into a redistribution of power or a transformation of traditional roles in the production of scientific knowledge, an aspect that lies at the core of citizen science [3, 12], it may represent an important step towards broadening participatory approaches in health research by consolidating practices of dialogue, trust, and recognition of lived experience.

Citizen science in health may contribute to a deeper understanding of population needs and to the promoting of solutions that are better aligned with diverse social contexts [1, 13]. However, important gaps remain regarding best practices, methodologies and strategies for evaluating its implementation in health research [3, 4, 14]. In this sense, the planning of CS initiatives in health should be informed by citizens’ perspectives in order to support realistic, inclusive and sustainable forms of involvement. This is particularly relevant because factors such as scientific and health literacy, perceptions of one’s role in the research process, and motivations for involvement may directly shape involvement and the quality of research partnerships [15–17].

Although involvement in health research varies by context, an increasing number of initiatives internationally have actively involved older adults in projects focused on eHealth, accessibility, age-friendly environments, and health planning. In these initiatives, older adults have contributed to data generation and the co-design of solutions for more inclusive communities [18–23]. Such experiences suggest the relevance of this age group as collaborators and point to possible benefits of participatory processes, including strengthened digital literacy, social empowerment, and the production of richer, more contextualised data [20, 24].

Nevertheless, studies remain scarce that examine, from older adults’ perspectives, the meanings attributed to involvement in health research, as well as motivations, perceived barriers, and support conditions for sustained participation. This gap is particularly relevant when involvement is still prospective, as older adults’ prior understandings and expectations may influence their willingness to engage, the roles they imagine for themselves, and the design of inclusive strategies. This study does not examine older adults’ actual involvement in a citizen science project; rather, it uses citizen science as a conceptual lens to understand how community-dwelling older adults perceive and anticipate prospective involvement in health research following community outreach activities. Against this backdrop, the aim of this exploratory study was to explore community-dwelling older adults’ perceptions and expectations regarding prospective involvement in health research through a citizen science lens.

## Methods

### Study design and setting

This was an exploratory, descriptive qualitative study reported in line with the Consolidated Criteria for Reporting Qualitative Research (COREQ) [25]. The study was conducted within a community outreach experience developed under Project X at Institution Y, in the health field, with a focus on promoting active ageing.

### Community outreach context

Initially, the research team contacted several health institutions in a municipality in Central Portugal to present the project and invite partnerships with local health teams. Following institutional agreement, the research team delivered five informational sessions as part of a community outreach initiative aimed at older adults. These sessions were attended by approximately 50 older adults who had been participating in an active and healthy ageing programme for around eight years. The sessions took place after the group’s regular activity sessions and addressed topics related to health promotion and health research, including mental health and ageing, nursing research, how research can help address everyday health problems, examples of ongoing projects, and ethical aspects of health research.

These sessions were not designed as formal citizen science training, but as an opportunity to foster dialogue, proximity and trust between the research team and the community. They also allowed participants to become familiar with basic ideas about health research and the possible role of citizens in research processes before being invited to reflect on prospective involvement in health research. This outreach context was considered appropriate because participants were already engaged in an active ageing programme but had no prior experience as citizen scientists.

### Participants and sampling

Eight community-dwelling older adults participated in the study. All were members of an active and healthy ageing programme, which provided a context of regular social interaction and potential interest in collaborative initiatives. Participants were recruited using non-probabilistic convenience sampling from among older adults who had attended the community outreach sessions. Inclusion criteria were: being aged 65 years or older, living in the community, having participated in the active ageing programme for at least six months, and being able to communicate in Portuguese. None of the participants had prior experience as citizen scientists.

At the end of the last community outreach session, the research team invited interested older adults to participate in a focus group. The purpose of the study, the voluntary nature of participation, confidentiality procedures and the possibility of withdrawing at any time were explained before written informed consent was obtained.

### Data collection procedures

Data were collected through a sociodemographic questionnaire and one focus group conducted in Portuguese chosen because it facilitates discussion and deeper exploration of topics through interaction among participants and researchers [26]. The focus group took place in person, in the facilities where the active and healthy ageing programme was held, after the community outreach activities described above. It lasted 76 min and was audio-recorded with participants’ consent.

The focus group was facilitated by E.S. and J.B.; J.B. also took field notes on group dynamics and non-verbal cues. A semi-structured guide was used to support the discussion, including open-ended questions about participants’ understandings of involvement in health research, perceived motivations, anticipated benefits, possible barriers, support needs, and preferred roles in future research initiatives. Examples of questions included: “What does being involved in health research mean to you?”, “How do you think older adults could contribute to health research?”, “What would motivate you to involve?”, “What difficulties might make involvement challenging?”, and “What support would be needed for older adults to become involved?”

### Data analysis

Audio recordings were transcribed verbatim and anonymised before analysis. Data were analysed using content analysis, following Bardin’s framework [27]. The analysis involved repeated reading of the transcript to become familiar with the material, identification of initial codes and meaning units, and subsequent organisation of these codes into subthemes and themes. ATLAS.ti software was used to support data organisation and coding.

Two researchers (E.S. and J.B.) were involved in the coding and interpretation process. Initial coding was discussed between the researchers, and the emerging analytical structure was refined through iterative comparison between the coded material, meaning units, subthemes and themes. Disagreements or uncertainties were resolved through discussion until consensus was reached. The final thematic structure was reviewed by the wider research team to enhance coherence and consistency with the study aim.

### Reflexivity and scientific rigour

The research team has experience in qualitative research, public health, and citizen science. To enhance the trustworthiness of the study, the focus group was audio-recorded and transcribed verbatim, and the analysis followed a systematic content analysis process supported by ATLAS.ti. Two researchers (E.S. and J.B.) discussed coding and theme development, and any discrepancies were resolved by consensus. The final thematic structure and interpretation of the findings were subsequently reviewed by the wider research team to strengthen coherence between the data, categories, and conclusions.

Given that the focus group followed previous community outreach sessions delivered by the research team, reflexivity was particularly important during the analysis. Although these sessions were not designed as formal citizen science training, prior contact with the researchers and discussion of health- and research-related topics may have influenced participants’ understandings and expectations. For this reason, the findings are interpreted as situated within this specific community outreach context.

### Ethical considerations

All ethical procedures were followed, ensuring anonymity, confidentiality, and the right to withdraw at any time. The study was approved by the Ethics Committee (Approval No. XX), and all participants provided written informed consent prior to participation.

Older adults were not involved as research partners in this study (i.e., in setting the research questions, designing the study, analysing the data, writing the manuscript, or facilitating the sessions). They participated as focus group participants, and two public contributors (one of whom was an older adult) reviewed and validated the Plain Language Summary.

## Findings

**Fig. 1:**
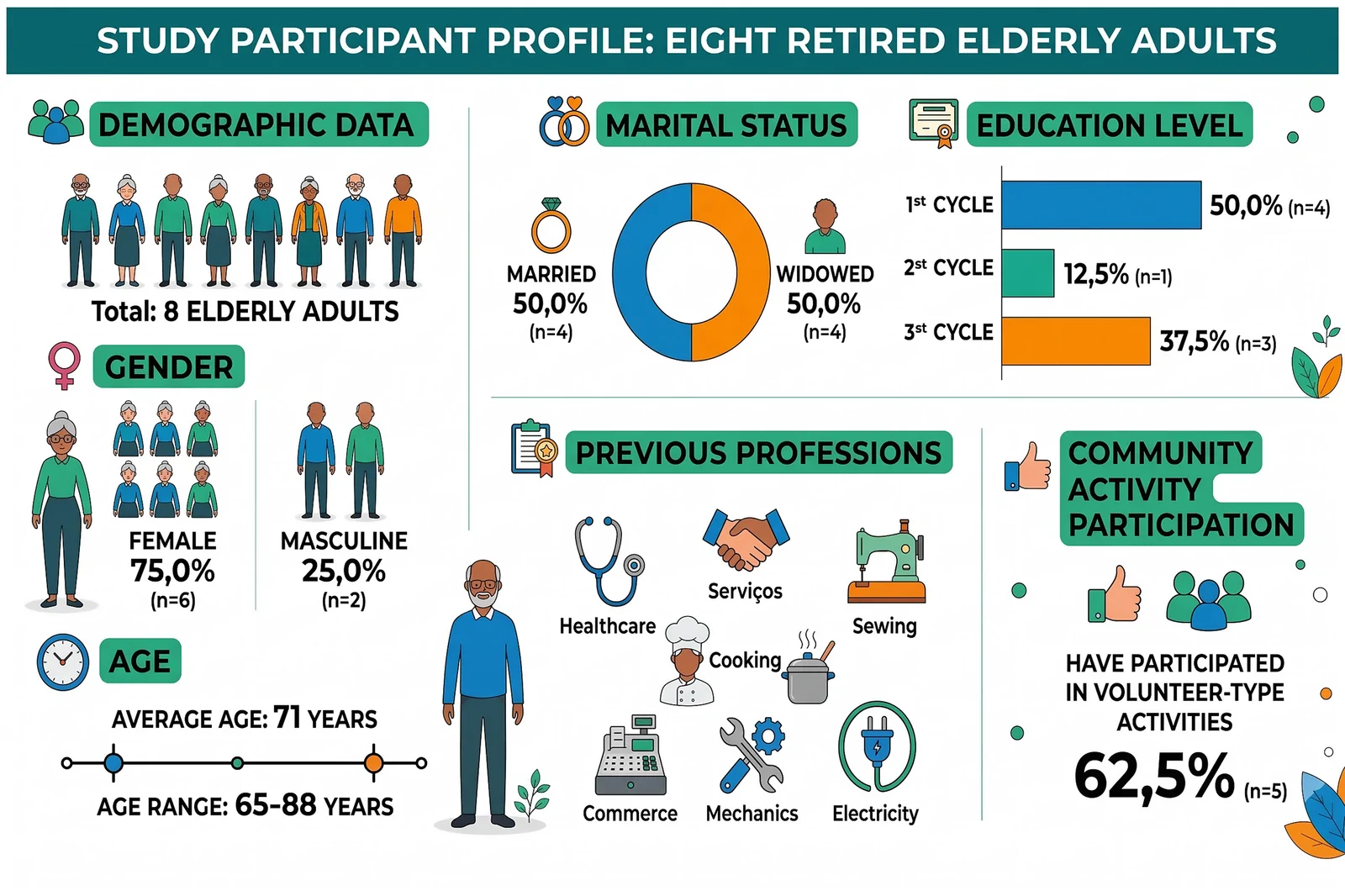
Characterization of the participants. Created by the authors with the aid of AI

Study participants were eight older adults, all retired; six were women (75.0%), with a mean age of 71 years (range: 65–88). Four participants were widowed (50.0%) and four were married (50.0%). Regarding educational attainment, four participants (50.0%) had completed primary education (1st cycle), one (12.5%) had completed lower secondary education (2nd cycle), and three (37.5%) had completed lower secondary education (3rd cycle). Participants’ previous occupations included health-related roles, services, sewing, cooking, retail, electrical work, and mechanics. Five participants (62.5%) reported prior involvement in community activities such as volunteering (Fig. 1).

The findings presented below should be understood as accounts of how participants perceived and anticipated prospective involvement in health research through a citizen science lens, rather than as reflections on lived experience as citizen scientists.

The meanings participants attributed to the topic allowed us to identify themes and subthemes highlighting the motivations, expected benefits and challenges associated with this prospective involvement (Table 1).

**Table 1:** Themes, subthemes, and meaning units related to citizens’ perceptions and experiences of their involvement in health research

Theme	Subtheme	Meaning units
1. Between volunteering and citizen science	Understanding involvement as volunteering	Volunteering in a school; support group for older adults
2. Becoming a future citizen scientist: identity, motivations, and perceived benefits	2.1 Personal motivations	Desire to feel useful; filling time in retirement; helping others
	2.2 Perceived benefits	Lifelong learning; strengthened citizenship; impact on health
	2.3 Self-perception and identity in involvement	Partner; collaborator
3. Becoming a future citizen scientist: challenges and necessary conditions for involvement	3.1 Training and ongoing support	Capacity-building and support from the research team
	3.2 Accessible communication	Difficulty with technical language; strategies to make communication clear
	3.3 Commitment and expectations	Flexibility vs. ongoing commitment; impact on personal routines

## Between volunteering and citizen science

The analysis of participants’ accounts shows that they strongly associate involvement in scientific research projects with volunteering. When asked to reflect on what “involvement in research” means, they spontaneously drew on prior experiences of community support particularly in schools and nursing homes suggesting that they primarily understand such involvement as a form of community service and solidarity.

Participants described these experiences as opportunities for direct contact with others, characterised by the provision of emotional support, awareness-raising, and knowledge-sharing, as illustrated in the following excerpts:

*I was involved in a project about drugs in schools*,* during adolescence. It aimed to encourage students to recognise drugs and say “no” to them. We would take one hour of class and give a presentation. The project*,* if I’m not mistaken*,* was called the “life project”. We worked in schools*,* mainly with lower and upper secondary students. We carried out activities so that they would become aware of the illusion created by drugs*,* alcohol*,* and tobacco. It was basically an awareness-raising activity. (P1)*

*A few years ago*,* I took part in a volunteering project at a school that involved educational activities. Teachers and students appreciated it. (P2)*

*I was part of a group that visited older people every Monday*,* every week… People in their 80s and 90s—many of them have since passed away. And we really enjoyed it*,* because they like to talk and to be with familiar people*,* to talk about things that sometimes we didn’t even know because they were from long ago. But it was very*,* very good. (P3)*

## Becoming a future citizen scientist: identity, motivations, and perceived benefits

This theme captures participants’ perceptions and expectations regarding potential involvement in scientific research projects. Although participants had no prior experience as citizen scientists, their accounts conveyed a strong sense of motivation and purpose associated with the idea of getting involved. The desire to contribute to society, stay active, and find meaning in their actions emerged as central drivers, alongside the belief that such involvement could generate both personal and collective benefits. In this way, expressed motivations were closely intertwined with anticipated benefits, highlighting the transformative potential of active involvement even before any direct experience takes place.

### Personal motivations

The personal motivations emerging in this subtheme suggest that, for participants, the possibility of collaborating in research projects is viewed positively—linked both to a social commitment to contribute to the common good and to the potential for personal benefit. Although they had not yet taken part in initiatives of this kind, participants anticipated the experience as an opportunity to find a renewed sense of purpose, particularly in retirement, valuing feelings of usefulness and the continuation of an active role in society.

In their accounts, their motivation for getting involved in scientific activities was also linked to making good use of their time, keeping their minds active and reducing social isolation issues often described as a challenge at this stage of life. As such, involvement in research was framed as a pathway to social connection and wellbeing, supporting not only collective contribution but also personal strengthening, as illustrated in the excerpts below:

*Yes*,* to fill our time in retirement—I’m speaking for myself. (P5)*

*But this is interesting… There’s always that wish to be able to help; nowadays it would be different—it’s about being useful. It’s a feeling many people must have*,* I think. I like being able to help others. (P2)*

*Because at our age we often become isolated. And I think it’s essential—for our mind*,* too—that we have companionship*,* talk… I’m not saying we only talk about our own problems*,* but the fact of talking and listening to other people’s problems… We feel we should help*,* and that… I think it’s good for us—we feel satisfied with that. It distracts us from our problems—not only older people*,* but also the others we help. (P4)*

*The point is—we just need to understand what matters to us. What motivates us. That’s the starting point. That’s the issue. (P8)*

### Perceived benefits

Within the discussions that emerged in this context, participants expressed broadly positive views about the possibility of becoming involved in activities that could support learning and social interaction. They perceived involvement in research projects as a valuable opportunity, associated with learning, social connection, and wellbeing.

This subtheme closely overlaps with the previous one, as the anticipated benefits of future involvement often mirrored the motivations participants described. For them, the possibility of becoming involved in scientific activities—whether through planning meetings or during project implementation—was perceived as a possible pathway to social connection and ongoing learning, which could help keep the mind active and strengthen a sense of purpose at this stage of life. Furthermore, participants recognised the importance of continuing to take part in initiatives with social relevance, particularly in the field of health, as a way that could support cognitive and physical stimulation, as illustrated in the excerpts below:

*There’s no age limit for learning. Everyone keeps learning*,* whether through this app (referring to a project on this topic) or in their daily lives. (P2)*

*It’s different*,* but it allows companionship because it will also foster connection with other people—that would be one aspect. (P1)*

*These contributions are important*,* especially the ones done manually*,* because besides making the mind think and develop—or*,* let’s say*,* not remain stagnant… (P4)*.

The idea of interacting with different generations, and the recognition that knowledge accumulated over a lifetime is valuable, were also described as rewarding. Participants viewed the opportunity to be involved not only as a means of contributing to society, but also as a chance for personal validation—helping them step away from negative thoughts and regain a sense of purpose:

*We may only have second grade*,* or third grade*,* or fourth grade*,* but we have knowledge in other areas that should be—and still isn’t—valued. (P6)*

*It makes them*,* at that moment—during that hour—fully absorbed in what they’re doing*,* rather than drifting and thinking about other things that sometimes make them feel*,* let’s say*,* more negative. (P8)*

### Self-perception and identity in involvement

Beyond motivations and anticipated benefits of future involvement, how citizens imagine they will be recognised and positioned within research projects appears to be a key factor shaping their willingness to get involved. When invited to reflect on the roles they could play and the contributions they might offer to science, participants used different labels for themselves, revealing varied perceptions of their identity and their place within the research process.

Terms such as “participant”, “collaborator”, “partner”, and “senior” were particularly valued because, in participants’ view, they convey active, respectful, and meaningful participation. These choices reflect a desire to be recognised as legitimate members of the research team, strengthening a sense of belonging and personal validation—elements that are fundamental for sustained involvement in citizen science initiatives, as illustrated in the excerpts below:

*Participant*,* collaborator… it’s good—it sounds nice. (P1)*

*Or “senior partner”—I like that as well. (P5)*

*Help from seniors*,* that’s it… partner. (P7)*

How participants see themselves—and wish to be seen—shapes the meaning they attribute to involvement, suggesting that symbolic recognition and a sense of belonging are central to sustaining commitment and effective involvement within a citizen science approach.

## Becoming a future citizen scientist: challenges and necessary conditions for involvement

This theme highlights, in a complementary way, the elements participants considered necessary for involving in scientific activities, as well as the potential challenges they anticipated.

### Training and ongoing support

Participants emphasised the need for adequate training and noted that appropriate guidance and supervision by experienced professionals would be essential to ensure they feel prepared for the tasks involved:

*Individually*,* I think each person should be assessed (…) Because everyone has capacity. So there has to be an evaluator. (P1)*

*Personally*,* I’d like to try… But I think it should be guided by someone experienced… (P2)*.

*Yes*,* but someone—so*,* guidance. Someone to supervise the project. And to keep (…) guiding (…) whether they have the ability*,* the training and the support (…) and keep guiding*,* right? (P2)*

Participants’ accounts reflect an understanding of involvement that remains strongly anchored in a traditional view of science, in which researchers are seen as the main holders of expertise and the primary drivers of the process. This perspective may be linked to the lack of prior experience in collaborative research contexts, leading participants to place particular value on expert oversight and ongoing support, while not yet fully recognising their own potential contribution.

Although this reflects a relatively early conception of involvement, the emphasis on training and technical support also signals a positive willingness to learn and participate in a more structured way, pointing to genuine interest in contributing to health research processes.

### Accessible communication

An important issue raised by participants concerned accessibility and communication within health research processes. Most participants described communication as a central challenge, noting that they often encounter terms they do not understand. They emphasised the need to simplify scientific language and make information easier to follow, particularly given the population’s varying levels of literacy, as illustrated in the excerpts below:

*Simplifying things—that’s important for people who don’t understand. Don’t use strange terms*,* because there is still a very large population… some people can’t even read. They also have to think about all citizens. (P8)*

*There needs to be an effort to make communication clearer. (P4)*

*Many times we don’t understand what they say. So there has to be an effort on both sides. (P2)*

*And that “R” (referring to the COVID-19 reproduction number) did anyone explain what it was? They said it was that “R”*,* and then we don’t understand… So what does it mean to simplify—to inform people*,* to support literacy. (P1)*

*I think that first*,* if we’re going to understand the reading of a book*,* we have to learn to read. And learning to read means understanding exactly those terms—what they mean. So there has to be something—some support*,* some training—before putting those strange terms out there. Otherwise only a few people understand. (P2)*

Overall, participants’ perspectives highlight scientific language as a substantial barrier to meaningful citizen involvement in research. Difficulties in understanding technical terms and specialised concepts reinforce the importance of clearer, more inclusive communication that can be adapted to different levels of health literacy. These concerns suggest that participants value transparency and knowledge sharing, and recognise access to understandable information as a prerequisite for active participation in citizen science initiatives.

### Commitment and expectations

Participants also highlighted the importance of considering their availability and expectations regarding their role in the process. In their accounts, they expressed interest in contributing, while emphasising that this future involvement needs to be compatible with their routines and other personal commitments:

*It requires availability. If it’s occasional—if I’m asked—I’m even willing to do it. But having a commitment… only if it were within a group*,* like: today you go*,* tomorrow it’s me*,* because today I can’t. Because if someone commits*,* they have to be available and always available. That’s a bit complicated. (P7)*

While some participants preferred more flexible, occasional involvement, others recognised that more regular commitment might be needed to ensure continuity and impact. Issues such as time availability, existing responsibilities, and expectations about their contribution emerged as key factors shaping both initial involvement and ongoing participation:

*It’s not because it’s volunteering that I agree I’ll be there today and then—oh*,* I’ve been invited to lunch. That also involves deciding whether to accept or withdraw*,* right? (…) Because*,* of course*,* we have our own lives too*,* don’t we? On Tuesdays and Thursdays there’s already exercise class. So if it clashes with that schedule*,* that’s already a factor for not taking part. (P5)*

Overall, participants’ accounts indicate that availability and the level of commitment required are decisive for both uptake and continuity. Although they expressed willingness to collaborate in the future, they also stressed the need to balance participation with personal routines and responsibilities. Involvement was therefore viewed positively, but as something that should respect individual limits and offer flexibility, enabling sustainable active participation that fits with citizens’ everyday lives.

## Discussion

This exploratory qualitative study examined the perceptions and expectations of eight community-dwelling older adults regarding prospective involvement in health research through a citizen science lens. Participants were not reporting lived experiences as citizen scientists; rather, they reflected on possible future roles, motivations, anticipated benefits, barriers and support conditions. In this sense, the study provides preliminary insights into how older adults anticipate involvement before participating in such initiatives, highlighting elements that may need to be clarified during the preparation and recruitment stages of future projects.

A central finding was that participants interpreted possible involvement in research through familiar repertoires of volunteering, usefulness and contribution to the common good. This association is understandable, as citizen science has historical links with voluntary participation in science and, in some frameworks, is described in terms of citizens’ voluntary contribution to research [5, 15, 28–32]. For older adults with no prior experience as citizen scientists, volunteering may therefore function as an accessible interpretive bridge for imagining future involvement. In the present study, however, participants framed involvement less as participation in the production of scientific knowledge and more as commitment to social causes, suggesting that the boundary between volunteering, social contribution and citizen science may not be immediately clear to potential participants [33].

At the same time, this framing requires careful attention. Although familiarity with volunteering may facilitate initial involvement by activating pre-existing dispositions towards collaboration, helping others and contributing to collective causes [34–37], it may also obscure citizens’ potential role in shaping research processes if involvement is presented mainly as “helping researchers” or supporting a social cause. Therefore, future citizen science initiatives may build on the motivational familiarity of volunteering, provided that they explicitly clarify the intended level of involvement, whether consultative, collaborative or co-creative, as well as the opportunities for citizens to influence the process and the expectations regarding their contributions [38]. This distinction is particularly important because volunteering may support initial involvement, but it should not replace explicit discussion of citizens’ roles in the production and communication of scientific knowledge.

Participants’ motivations for becoming involved in health research and the benefits they anticipated from such involvement were closely intertwined. They associated prospective involvement with contributing to the common good, lifelong learning, maintaining cognitive activity, social connection and feeling useful. These motivations are consistent with previous research showing that citizens may be motivated by personal interest, learning opportunities, social interaction, a sense of belonging and contribution to meaningful causes [16, 35, 39–43]. In health-related contexts, enthusiasm for the topic and the perception that one’s actions may contribute to tangible change have also been highlighted [44].

For these participants, prospective involvement in health research therefore appeared meaningful not only because it could support the project, but also because it could offer opportunities for learning, social connection and feeling useful. This emphasis on intrinsic motivations, such as personal interest and satisfaction from contributing to society, is also consistent with previous European research suggesting that older adults may be particularly motivated by non-material incentives [45].

These motivations were also linked to how participants imagined their future role in research projects. By seeing involvement as a way to contribute, learn and feel useful, participants positioned themselves not only as people taking part in a study, but potentially as collaborators or partners in the research process. In this sense, terms such as “collaborator” or “partner” were not merely labels; they indicated how participants imagined their place in future research projects. The terminology used to describe citizens’ roles matters because it may influence how they understand their place in the research process and whether they perceive themselves as legitimate contributors to knowledge production [15, 28, 29]. Previous studies suggest that recognition may support commitment, continuity and a sense of belonging in citizen science initiatives [15, 44, 46, 47].

In this study, the relevance of recognition emerged prospectively: participants valued the possibility of having their contributions acknowledged and of being recognised as more than study participants. Examples from previous initiatives, including the use of “Health Ambassador” certificates or the explicit acknowledgement of citizen scientists’ contributions, illustrate how recognition can help make involvement more visible and meaningful [17, 44]. This is relevant because recognition should not be understood only as a symbolic gesture, but also as part of how citizens’ experiential and community knowledge is positioned within the research process. In this regard, the findings resonate with broader concerns about tokenism and epistemic injustice in CS [2, 48], especially when citizens are invited to contribute but have limited influence over how their knowledge is used. For future projects in health involving older adults, clarifying roles and forms of recognition may therefore be important not only for recruitment, but also for sustaining involvement over time.

Participants also identified several conditions that may support future involvement in health research through a citizen science lens, including accessible communication, training, ongoing support, flexibility and clear alignment of expectations. These conditions are widely recognised in citizen science and participatory research literature and should not be interpreted as entirely new barriers [15, 44, 49]. Their relevance in this study lies in the fact that they were identified before actual involvement, suggesting important aspects that researchers may need to consider not only during recruitment, preparation and role negotiation, but also throughout the involvement process.

Accessible language appeared particularly important, as technical terminology and scientific jargon may reinforce distance between researchers and citizens and make involvement less inclusive [15, 17, 44, 50, 51]. This supports the need for plain language, culturally adapted concepts and information provided through different formats and communication channels [4, 17, 51–54].

Training and regular contact with the research team were also seen as ways to build trust, support confidence and clarify tasks. This is particularly relevant because participants were speaking from the standpoint of people who had not yet been involved in citizen science projects in health and who therefore anticipated that limited familiarity with research processes, low confidence in performing research-related tasks, or uncertainty about their active role could hinder their involvement [4, 49–53, 55].

Finally, flexibility regarding time, availability and personal circumstances appeared necessary to make involvement compatible with everyday routines. Early alignment around project aims, expected contributions, stages of the process and implementation timelines may also help sustain involvement over time [55, 56]. In this sense, expectations around citizen involvement should be balanced with strategies that support a sustained and meaningful role throughout the process.

Although citizen involvement in this study was limited to contributing to the Plain Language Summary (PLS), the findings may inform the design of future initiatives aiming to involve older adults more effectively as research partners.

Despite the contributions of this study to understanding older adults’ involvement in citizen science in health, several limitations should be considered. The study was based on a single focus group with eight community-dwelling older adults, all recruited from a specific active ageing programme in Central Portugal and with no prior experience as citizen scientists. This specific context should be considered when interpreting the findings.

The previous community outreach sessions and prior contact with the research team may have shaped participants’ understandings and expectations. Therefore, the findings should be interpreted as preliminary and situated insights, rather than as directly transferable to older adults in different communities, health systems or cultural contexts without careful consideration of context.

Future studies should include older adults with practical experience in citizen science, compare different institutional and community contexts, and examine longitudinally how involvement is sustained over time and how citizens’ roles evolve throughout the research process.

## Conclusion

This exploratory study provides preliminary and situated insights into community-dwelling older adults’ perceptions and expectations regarding prospective involvement in health research through a citizen science lens. Participants understood possible involvement through familiar ideas of volunteering, usefulness and contribution to the common good, while also associating it with learning, social connection, maintaining cognitive activity and feeling useful. These findings suggest that, even before becoming involved in citizen science initiatives, participants recognised their potential to contribute experiences, perspectives and community knowledge, while also anticipating the need for clear roles, accessible communication, training, support, flexibility and recognition to make such involvement feasible and meaningful.

From a practical perspective, these findings point to the need to make CS more visible and better understood among older adults, particularly in the field of health. The participants’ association between citizen science and volunteering may offer an accessible entry point for involvement, as it connects research with familiar ideas of contribution, usefulness and the common good. However, this proximity must be approached with clarity and honesty. Researchers should avoid reducing involvement to an invitation to simply “help” the project and should instead make explicit how citizens’ experiences, perspectives and community knowledge can inform the research process. This is important to avoid tokenistic forms of involvement and to reduce the risk that citizens’ experiential and community knowledge is invited without being genuinely valued within the research process. In this sense, accessible communication, training, ongoing support, flexibility, recognition and clear negotiation of roles and expectations may help reduce uncertainty and support older citizens in assuming a more confident and meaningful role throughout the process.

At an institutional level, these findings point to the importance of creating conditions that make citizens’ contributions visible, valued and clearly connected to the research process. Future studies should include older adults with practical experience in citizen science, compare different institutional and community contexts, and examine longitudinally how involvement is sustained over time, how citizens’ roles evolve, and how institutional models or public policies shape opportunities for meaningful involvement in health research.

## Supplementary Information

Below is the link to the electronic supplementary material.

Supplementary Material 1

Supplementary Material 2

## Data Availability

The datasets generated and/or analysed during the current study are not publicly available due to the sensitive nature of the data and participant confidentiality. They are available from the corresponding author on reasonable request.

## References

[1] Den Broeder L, Devilee J, Van Oers H, Schuit AJ, Wagemakers A. Citizen science for public health. Health Promot Int. 2018;33(3):505–14. 10.1093/heapro/daw086. PMC6005099 28011657

[2] Groot B, Abma T. Ethics framework for citizen science and public and patient participation in research. BMC Med Ethics. 2022;23(1):23. 10.1186/s12910-022-00761-4. PMC8919534 35282819

[3] Remmers G, Tzovaras BG, Albert A, Van Laer J, Wildevuur S, De Groot M, et al. Citizen science for health: an international survey on its characteristics and enabling factors. Citiz Sci Theory Pract. 2024;9(1):23. 10.5334/cstp.693.

[4] Rowbotham S, Walker P, Marks L, Irving M, Smith BJ, Laird Y. Building capacity for citizen science in health promotion: a collaborative knowledge mobilisation approach. Res Involv Engagem. 2023;9(1). 10.1186/s40900-023-00451-4. PMC10228445 37254184

[5] Vohland K, Land-Zandstra A, Ceccaroni L, Lemmens R, Perelló J, Ponti M, et al. editors. The science of citizen science. Cham: Springer; 2021. 10.1007/978-3-030-58278-4.

[6] Haklay MG, Huyse M, Paleco T, Stokart C, Mazzonetto L, Radicchi A. Mutual learning exercise on citizen science initiatives: policy and practice. First topic report: introduction and overview of citizen science (Horizon Europe Policy Support Facility). Luxembourg: Publications Office of the European Union; 2022. 10.2777/29886.

[7] King AC, Winter SJ, Chrisinger BW, Hua J, Banchoff AW. Maximizing the promise of citizen science to advance health and prevent disease. Prev Med. 2019;119:44. 10.1016/j.ypmed.2018.12.016. PMC6687391 30593793

[8] Kullenberg C, Kasperowski D. What is citizen science? A scientometric meta-analysis. PLoS ONE. 2016;11(1):e0147152. 10.1371/journal.pone.0147152. PMC4713078 26766577

[9] Luís C. A Ciência Cidadã: passado, presente e futuro do envolvimento público na investigação científica. Rev Lusófona Estud Cult. 2022;9(2):29–42. 10.21814/rlec.4051.

[10] Heyen NB, Gardecki J, Eidt-Koch D, Schlangen M, Pauly S, Eickmeier O, et al. Patient science: citizen science involving chronically ill people as co-researchers. J Particip Res Methods. 2022;3(1). 10.35844/001c.35634.

[11] Rosas LG, Espinosa PR, Jimenez FM, King AC. The role of citizen science in promoting health equity. Annu Rev Public Health. 2021;43(1). 10.1146/annurev-publhealth-090419-102856. PMC9034747 34724389

[12] Hammel G, Woll S, Baumann M, Scherz C, Maia M, Behrisch B, et al. Bürgerwissenschaftliche Forschungsansätze in Medizin und Gesundheitsforschung: ausgewählte Begriffe mit Fokus auf den Beteiligungsgrad. TATuP. 2021;30(3):63–9.

[13] Silva RCG, Santana ES. Ciência cidadã. Cogitare Enferm. 2023;28:e86901. 10.1590/ce.v28i0.86901.

[14] Borda A, Gray K, Downie L. Citizen science models in health research: an Australian commentary. Online J Public Health Inf. 2019;11(3):e23. 10.5210/ojphi.v11i3.10358. PMC6975539 31993110

[15] Eitzel MV, Cappadonna JL, Santos-Lang C, Duerr RE, Virapongse A, West SE, et al. Citizen science terminology matters: exploring key terms. Citiz Sci Theory Pract. 2017;2(1):1. 10.5334/cstp.96.

[16] Hano MC, Wei L, Hubbell B, Rappold AG. Scaling up: citizen science engagement and impacts beyond the individual. Citiz Sci Theory Pract. 2020;5(1):1. 10.5334/cstp.244. PMC7529103 33014428

[17] Silva SMDT, Santana ES, Bernardo JVR, Silva AC, Lopes C, Almeida CV, et al. A ciência cidadã na saúde para coconstrução de sumários de evidência para o cidadão. Rev Enferm Ref. 2025;6(4):1–8. 10.12707/RVI24.96.36966.

[18] Bernardo J, Santana E, Silva S, Barroco de Melo E, Apóstolo J, Simões M et al. Digital health for aging in place: a co-design study. Games Health J. 2026. 10.1177/2161783X261445292. 42099120

[19] Frögren J, Granbom M, Jonsson O, Bergman M, Iwarsson S. Characteristics and attitudinal changes of senior citizens and persons with functional impairments involved in a citizen science project on housing accessibility. Citiz Sci Theory Pract. 2023;8(1):57. 10.5334/cstp.551.

[20] King AC, King DK, Banchoff A, Solomonov S, Ben Natan O, Hua J, et al. Employing participatory citizen science methods to promote age-friendly environments worldwide. Int J Environ Res Public Health. 2020;17(5):1541. 10.3390/ijerph17051541. PMC7084614 32121001

[21] McNeil H, Elliott J, Huson K, Ashbourne J, Heckman G, Walker J, et al. Engaging older adults in healthcare research and planning: a realist synthesis. Res Involv Engagem. 2016;2. 10.1186/s40900-016-0022-2. PMC5611557 29062511

[22] Porter M, Odeyemi E, Chesser S, King A. A citizen science project engaging Nigerian older persons in a neighborhood assessment for physical activity. Innov Aging. 2024;8:524–524. 10.1093/geroni/igae098.1714. PMC10993715 38577518

[23] Wood GER, Pykett J, Banchoff A, King AC, Stathi A, et al. Employing citizen science to enhance active and healthy ageing in urban environments. Health Place. 2023;79:102954. 10.1016/j.healthplace.2022.102954. 36493495

[24] Aristeidou M, Herodotou C. Online citizen science: a systematic review of effects on learning and scientific literacy. Citiz Sci Theory Pract. 2020;5(1):1–12.

[25] Tong A, Sainsbury P, Craig J. Consolidated criteria for reporting qualitative research (COREQ): a 32-item checklist for interviews and focus groups. Int J Qual Health Care. 2007;19(6):349–57. 10.1093/intqhc/mzm042. 17872937

[26] Backes D, Colomé J, Erdmann R, Lunardi V. O grupo focal como técnica de coleta de dados na pesquisa qualitativa. O Mundo da Saúde. 2011;35(4):438–42.

[27] Bardin L. Análise de conteúdo. São Paulo: Edições 70; 2022.

[28] Strasser B, Baudry J, Mahr D, Sanchez G, Tancoigne E. Citizen science? Rethinking science and public participation. 2019.

[29] Ngo KM, Altmann CS, Klan F. How the general public appraises contributory citizen science: factors that affect participation. Citiz Sci Theory Pract. 2023;8(1):3. 10.5334/cstp.502.

[30] West S, Pateman R. Recruiting and retaining participants in citizen science: what can be learned from the volunteering literature? Citiz Sci Theory Pract. 2016;1(2):15. 10.5334/cstp.8.

[31] Wiggins A, Wilbanks J. The rise of citizen science in health and biomedical research. Am J Bioeth. 2019;19(8):3–14. 10.1080/15265161.2019.1619859. 31339831

[32] Haklay M. Citizen science and volunteered geographic information: overview and typology of participation. In: Sui D, Elwood S, Goodchild M, editors. Crowdsourcing geographic knowledge. Dordrecht: Springer; 2013. 10.1007/978-94-007-4587-2_7.

[33] Haklay M. Participatory citizen science. In: Haklay M, Hecker S, Bowser A, Makuch Z, Vogel J, Bonn A, editors. Citizen science: innovation in open science, society and policy. London: UCL Press; 2018. p. 52–62. Available from: http://www.jstor.org/stable/j.ctv550cf2.11.

[34] Levontin L, Gilad Z, Shuster B, Chako S, Land-Zandstra A, Lavie-Alon N, et al. Standardizing the assessment of citizen scientists’ motivations: a motivational goal-based approach. Citiz Sci Theory Pract. 2022;7(1). 10.5334/cstp.459.

[35] Land-Zandstra A, Agnello G, Gültekin YS. Participants in citizen science. In: Vohland K, Land-Zandstra A, Ceccaroni L, et al. editors. The science of citizen science. Cham: Springer; 2021. 10.1007/978-3-030-58278-4_13.

[36] Allf BC, Cooper CB, Larson LR, Dunn RR, Futch SE, Sharova M, et al. Citizen science as an ecosystem of engagement: implications for learning and broadening participation. Bioscience. 2022;72(7):651–63. 10.1093/biosci/biac035. PMC9236874 35769502

[37] Waugh JK, Lindsey JK, Stewart MZ, Winter JC, Parrish JK. Demographics of public participation in science: a meta-analytic approach. Citiz Sci Theory Pract. 2023;8(1):61. 10.5334/cstp.610.

[38] Martin V. Citizen science as a means for increasing public engagement in science. Sci Commun. 2017;39:142–68. 10.1177/1075547017696165.

[39] Clark M, Cornes M. Citizen science as a framework for improving the science-society interface in long-term care research. J Long-Term Care. 2023;2023:92–9.

[40] Haywood BK. A sense of place in public participation in scientific research. Sci Educ. 2014;98(1):64–83. 10.1002/sce.21087.

[41] Lehman E, Jepson R, McAteer J, Archibald D. What motivates volunteers to engage in health-related citizen science initiatives? A case study of Our Outdoors. Int J Environ Res Public Health. 2020;17(19):6950. 10.3390/ijerph17196950. PMC7578925 32977509

[42] Levontin L, Gilad Z, Shuster B, Chako S, Land-Zandstra A, Lavie-Alon N, et al. Standardizing the assessment of citizen scientists’ motivations: a motivational goal-based approach. Citiz Sci Theory Pract. 2022;7(1). 10.5334/cstp.459.

[43] Pelacho M, Orejudo S, Clemente-Gallardo J. Science as a commons: motivations for continued participation in citizen science projects. PLoS ONE. 2025;20(6):e0325593. 10.1371/journal.pone.0325593. PMC12192172 40560832

[44] Den Broeder L, Lemmens L, Uysal S, Kauw K, Weekenborg J, Schönenberger M, et al. Public health citizen science: perceived impacts on citizen scientists—A case study in a low-income neighbourhood in the Netherlands. Citiz Sci Theory Pract. 2017;2(1):7. 10.5334/cstp.89.

[45] Lakomý M, Hlavová R, Machackova H, Bohlin G, Lindholm M, Bertero MG, et al. The motivation for citizens’ involvement in life sciences research is predicted by age and gender. PLoS ONE. 2020;15(8):e0237140. 10.1371/journal.pone.0237140. PMC7398498 32745131

[46] Santana E, Cardoso AF, Bernardo J, Silva S, Santos D, Ventura F, et al. Science communication for citizens: speeches in use. New Trends Qual Res. 2022;15:e745. 10.36367/ntqr.15.2022.e745.

[47] Santana E, Bernardo J, Donici I, Valente R, Pedro B, Almeida I, et al. An analysis of science communication about COVID-19 vaccination in Portuguese online news media. JCOM. 2023;22(05):A02. 10.22323/2.22050202.

[48] Herzog L, Lepenies R. Citizen science in deliberative systems: participation, epistemic injustice, and civic empowerment. Minerva. 2022;60(4):489–508. 10.1007/s11024-022-09467-8. PMC9080978 35574292

[49] Aylward BL, Milford KM, Storey KE, Nykiforuk CIJ, Raine KD. Citizen science in monitoring food environments: a qualitative collective case study of stakeholders’ experiences during the Local Environment Action on Food project in Alberta, Canada. BMC Public Health. 2022;22(1). 10.1186/s12889-022-13030-1. PMC8985336 35387614

[50] Barrie H, Soebarto V, Lange J, McCorry-Breen F, Walker L. Using citizen science to explore neighbourhood influences on ageing well: pilot project. Healthc (Basel). 2019;7(4):126. 10.3390/healthcare7040126. PMC6956060 31683757

[51] Laird Y, Manner J, Baldwin L, Hunter R, McAteer J, Rodgers S, et al. Stakeholders’ experiences of the public health research process: time to change the system? Health Res Policy Syst. 2020;18:1–10. 10.1186/s12961-020-00599-5. PMC7368787 32682426

[52] Okop KJ, Kedir K, Kasenda S, et al. Multi-country collaborative citizen science projects to co-design cardiovascular disease prevention strategies and advocacy: findings from Ethiopia, Malawi, Rwanda, and South Africa. BMC Public Health. 2023;23(1). 10.1186/s12889-023-17393-x. PMC10714547 38087240

[53] Lewandowski E, Caldwell W, Elmquist D, Oberhauser K. Public perceptions of citizen science. Citiz Sci Theory Pract. 2017;2(1):3. 10.5334/cstp.77.

[54] Golumbic YN, Baram-Tsabari A, Koichu B. Engagement and communication features of scientifically successful citizen science projects. Environ Commun. 2020;14(4):465–80. 10.1080/17524032.2019.1687101.

[55] Winter SJ, Goldman Rosas L, Padilla Romero P, Sheats JL, Buman MP, Baker C, et al. Using citizen scientists to gather, analyze, and disseminate information about neighborhood features that affect active living. J Immigr Minor Health. 2016;18(5):1126–38. 10.1007/s10903-015-0241-x. PMC4715987 26184398

[56] Lorentzen C, Stang I. How to facilitate long-term citizen participatory processes as part of local public health work. Eur J Public Health. 2020;30(Suppl 5). 10.1093/eurpub/ckaa166.1176.

