# Supplementary material for: What do older adults think about involvement in health research through a citizen science lens? A situated qualitative study
Source: Res Involv Engagem. 2026 Oct 30;12:176. doi: 10.1186/s40900-026-00960-y (PMC13723125; doi:10.1186/s40900-026-00960-y)
Supplement: Supplementary file 1 [file 40900_2026_960_MOESM1_ESM.docx]

**Supplementary Material GRIPP2 Reporting Checklist - short form**

| Section and topic | Item | Reported on page |
| --- | --- | --- |
| 1) Aim | Report the aim of PPI in the study | Public involvement in this study aimed to improve the clarity, accessibility, and appropriateness of the Plain Language Summary (PLS) for non-academic readers. Pg 7,8 |
| 2) Methods | Provide a clear description of the methods used for PPI in the study | Two public contributors, one of whom was an older adult, reviewed and validated the Plain Language Summary (PLS). Their feedback focused on simplifying wording, improving clarity of key messages, and ensuring the text was appropriate for the intended audience. Pg 2,3 |
| 3) Study results | Outcomes—Report the results of PPI in the study, including both positive and negative outcomes | Feedback from the public contributors led to revisions to the PLS (e.g., removing/replacing technical terms, using shorter sentences, and clarifying key points), resulting in a clearer and more accessible final version. |
| 4) Discussion and conclusions | Outcomes—Comment on the extent to which PPI influenced the study overall. Describe positive and negative effects | Public involvement was limited to the dissemination stage (PLS). No older adults were involved as research partners in setting the research questions, designing the study, analysing the data, writing the manuscript, or facilitating the sessions; older adults participated as focus group participants. |
| 5) Reflections/critical perspective | Comment critically on the study, reflecting on the things that went well and those that did not, so others can learn from this experience | PLS validation reinforced the importance of clear, accessible communication to support meaningful involvement. As a limitation, public involvement did not occur in earlier stages of the research process. Future work should involve older adults as partners beyond dissemination, including in setting priorities and refining recommendations. |
