# Supplementary material for: What do older adults think about involvement in health research through a citizen science lens? A situated qualitative study
Source: Res Involv Engagem. 2026 Oct 30;12:176. doi: 10.1186/s40900-026-00960-y (PMC13723125; doi:10.1186/s40900-026-00960-y)
Supplement: Supplementary file 2 [file 40900_2026_960_MOESM2_ESM.docx]

**COREQ (COnsolidated criteria for REporting Qualitative research) Checklist**

A checklist of items that should be included in reports of qualitative research. You must report the page number in your manuscript where you consider each of the items listed in this checklist. If you have not included this information, either revise your manuscript accordingly before submitting or note N/A.

| **Item COREQ** | **Guide Questions** | **Description** |
| --- | --- | --- |
| 1. Interviewer | Which author/s conducted the interview or focus group? | The focus group was led by E.S. and J.B. Pg 6 |
| 2. Credentials | What were the researcher’s credentials? | E.S.; PhD, J.B.: PhD Student |
| 3. Occupation | What was their occupation at the time of the study? | At the time of the study, E.S. and J.B. were researchers at a health sciences research unit |
| 4. Gender | Was the researcher male or female? | The facilitators were women (E.S. and J.B.). |
| 5. Experience and training | What experience or training did the researcher have? | The team had experience in qualitative research; the facilitators had experience and training in conducting focus groups. Pg 7 |
| 6. Relationship established | Was a relationship established prior to study commencement? | Yes. There had been prior contact during the informational sessions held before the focus group Pg 6 |
| 7. Participant knowledge of the interviewer | What did the participants know about the researcher? e.g. personal  goals, reasons for doing the research | Participants were aware that the researchers were conducting a study on citizen involvement in health research and that participation was voluntary and would not affect their access to the programme or health service. |
| 8. Interviewer characteristics | What characteristics were reported about the inter viewer/facilitator? e.g. Bias, assumptions, reasons and interests in the research topic | The team acknowledged potential contextual influences and encouraged self-reflection (post-session summary notes and analytical discussion) to mitigate biases. Pg 7 |
| 9. Methodological orientation and Theory | What methodological orientation was stated to underpin the study? e.g. grounded theory, discourse analysis, ethnography, phenomenology, content analysis | Exploratory qualitative study; focus group; content analysis (Bardin, 2022), supported by ATLAS.ti; reporting guided by COREQ. Pg 5 |
| 10. Sampling | How were participants selected? e.g. purposive, convenience, consecutive, snowball | Participants were recruited using non-probabilistic convenience sampling Pg 5,6 |
| 11. Method of approach | How were participants approached? e.g. face-to-face, telephone, mail,  email | In-person invitation, following the informational sessions, extended by researchers and healthcare professionals (nurses) from the field. Pg 6 |
| 12. Sample size | How many participants were in the study? | Eight participants Pg5 |
| 13. Non-participation | How many people refused to participate or dropped out? Reasons? | N/A |
| 14. Setting of data collection | Where was the data collected? e.g. home, clinic, workplace | Facilities of the healthcare institution where the program was held (in-person) Pg 6 |
| 15. Presence of nonparticipants | Was anyone else present besides the participants and researchers? | No. Only participants and researchers. |
| 16. Description of sample | What are the important characteristics of the sample? e.g. demographic data, date | Age, gender, marital status, education level, previous occupation; prior volunteer experience (Descriptive chart) Pg 8 |
| 17. Interview guide | Were questions, prompts, guides provided by the authors? Was it pilot  tested? | Semi-structured interview with guiding questions; There was no formal pilot study. Pg 6 |
| 18. Repeat interviews | Were repeat inter views carried out? If yes, how many? | N/A |
| 19. Audio/visual recording | Did the research use audio or visual recording to collect the data? | Yes, audio recording with consent |
| 20. Field notes | Were field notes made during and/or after the inter view or focus group? | Yes. J.B. took notes and observed nonverbal cues during the session. |
| 21. Duration | What was the duration of the inter views or focus group? | 76 minutes Pg 6 |
| 22. Data saturation | Was data saturation discussed? | Saturation was not formally assessed, given the exploratory design involving a single focus group; the results should be interpreted in context |
| 23. Transcripts returned | Were transcripts returned to participants for comment and/or correction? | N/A |
| 24. Number of data coders | How many data coders coded the data? | Two coders (E.S. and J.B.). |
| 25. Description of the coding tree | Did authors provide a description of the coding tree? | Yes. Theme–subtheme structure (presented in Table 1). |
| 26. Derivation of themes | Were themes identified in advance or derived from the data? | Inductively derived from the data |
| 27. Software | What software, if applicable, was used to manage the data? | ATLAS.ti. |
| 28. Participant checking | Did participants provide feedback on the findings? | N/A |
| 29. Quotations presented | Were participant quotations presented to illustrate the themes/findings? Was each quotation identified? e.g. participant number | Yes. Excerpts included and identified by participant (P#) Pg10-17 |
| 30. Data and findings consistent | Was there consistency between the data presented and the findings? | Yes. The topics are supported by excerpts and consistent with the analysis |
| 31. Clarity of major themes | Were major themes clearly presented in the findings? | Yes. Three main topics, each with subtopics |
| 32. Clarity of minor themes | Is there a description of diverse cases or discussion of minor themes? | Yes. Variation described within subthemes (e.g., flexibility vs ongoing commitment). |
